# Effects of age in a cognitive-motor dual task: parametric manipulations of cognitive load and motor difficulty

**DOI:** 10.3389/fnhum.2026.1927089

**Published:** 2026-09-23

**Authors:** C. M. Denaro, J. A. Monroy, A. A. Hartley, J. Asta, C. L. Reed

**Affiliations:** 1 Department of Psychological Science, Claremont McKenna College, Claremont, CA, United States; 2 Department of Natural Sciences, Claremont Colleges, Claremont, CA, United States

**Keywords:** aging, cognitive load, dual task, executive function, kinematics, motion capture, motor load, posture prioritization theory

## Abstract

**Introduction:**

Everyday mobility requires the continuous allocation of limited executive resources between cognitive and motor control. Prioritization theory predicts that biomechanical stability is prioritized as load increases to reduce fall risk. Although dual-task walking has been widely studied, most investigations examine the kinematic effects of a single cognitive task on a single walking condition, providing limited insight into how cognitive-motor interactions evolve as task demands progressively increase. Here we examined how increasing cognitive load and motor difficulty jointly influence gait adaptation in healthy younger and older adults.

**Methods:**

Younger (*n* = 29) and older adults (*n* = 30) performed auditory 0-, 1-, and 2-back tasks while walking on a treadmill at three inclines (level, intermediate, highest). Working memory performance and gait kinematics were analyzed.

**Results:**

Increased cognitive load produced declines in *d*′ and increases in RTs. Both age groups showed similar increases in RT with increased load, but older adults exhibited greater decreases in d′ with increased load. Age-related gait differences were observed, especially with increases in motor difficulty, but they were parameter-specific, concentrated in stride length, stride frequency and toe clearance rather than reflecting a uniform age-related decline across all gait measures. Compared with younger adults, older adults’ gait was characterized by lower toe clearances, higher stride frequencies, shorter relative stride lengths, and greater time in double support. As motor difficulty increased, younger adults progressively adapted their gait across all incline levels, whereas older adults made these adaptations only between the lowest and higher inclines. RT and *d*′—both presumed to reflect executive function—were related to gait in qualitatively different ways. RT was generally associated with gait parameters, while *d*′ was highly specific to incline, and in one case, task difficulty.

**Discussion:**

These findings support capacity-sharing and posture-first accounts of cognitive-motor interaction. In addition to age-related baseline differences, executive function explains additional variance in adaptive gait responses under increasing task demands.

## Introduction

Falling is the leading cause of injury and injury-related deaths in adults over the age of 65 (Dillon et al., 2010; Kakara et al., 2023a,b). As people age, balance and stability decline primarily due to muscle loss, sensory impairment and sensorimotor integration (Wang et al., 2024). In addition, older adults exhibit slower walking gaits with shorter step and stride lengths, a longer stance phase, and larger step widths compared to younger adults (Boyer et al., 2017). Cognitive processes are also integral to the control of walking, and age-related declines in cognition may compromise gait stability and adaptability in older adults. Falls frequently result from interactions between these age-related changes in gait and cognition and variation in the environment such as uneven terrain and unexpected obstacles (Barolo et al., 2026). Fall risk further increases when performing cognitive and motor tasks simultaneously, such as walking and talking (Ayers et al., 2014).

Numerous studies on cognitive–motor dual-task interference have demonstrated that cognitive effort interferes with walking and that increasing motor demands interfere with cognition (for reviews, see Al-Yahya et al., 2011; Beurskens and Bock, 2012; Mustafovska et al., 2025; Ramírez and Gutiérrez, 2021; Smith et al., 2017; Wollesen and Voelcker-Rehage, 2014; Wollesen et al., 2019; Woollacott and Shumway-Cook, 2002; Schaefer, 2014). When there is competition for attentional resources, an individual must switch between tasks and prioritize one task over the other (Pashler, 1994) or share the limited resources (Wickens, 2008) resulting in performance changes in either or both of the concurrent tasks. In general, healthy individuals prioritize gait stability over success of a cognitive task to maximize balance and minimize fall risk (Yogev-Seligmann et al., 2012). Walking, once initiated, can be automatic, but draws on attention, executive function and working memory in motorically difficult environments, particularly in older adults (Clark, 2015). Research by Clark (2015) confirms that healthy individuals naturally adopt a “posture-first” strategy during dual-task performance. When faced with competing cognitive and motor demands, they deliberately prioritize gait stability and balance to minimize fall risk, at the expense of their performance on the cognitive task. Thus, task prioritization depends on the relative demands of the motor and cognitive tasks, the available resources and individual characteristics, such as age, emotional state, and prior experience (Yogev-Seligmann et al., 2012).

A large body of research demonstrates that aging is associated with greater difficulty performing two tasks concurrently, even when neither task is especially demanding when performed alone. For example, older adults have difficulty coordinating multiple streams of information and managing task priorities, even when individual task requirements are relatively modest (Maquestiaux and Ruthruff, 2021). Further, older adults often show larger dual-task costs than younger adults across a wide variety of task combinations, including cognitive–cognitive, motor–motor, and cognitive–motor paradigms (Hartley, 2001; Hartley et al., 2015; Verhaeghen et al., 2003). These age differences have been attributed to declines in attentional resources, reduced processing efficiency, and age-related impairments in executive control processes responsible for coordinating concurrent activities (Kramer and Madden, 2008; Glass et al., 2000; Hartley, 2001). Glass et al. (2000) reported that older adults exhibited disproportionate costs when required to divide attention between concurrent activities, suggesting age-related limitations in task scheduling and attentional allocation. Hartley and Little (1999) demonstrated that older adults are less able than younger adults to flexibly allocate attention across competing tasks, leading to larger performance decrements under dual-task conditions. Although the precise mechanisms are debated, contemporary accounts generally converge on the view that dual-task performance depends heavily on executive-control processes that coordinate task priorities, schedule responses, and resolve competition for limited cognitive resources, all of which show age-related decline (Kramer and Madden, 2008; Maquestiaux and Ruthruff, 2021). Cognitive-motor dual-task paradigms provide a valuable context for examining these age-related limitations because successful locomotion increasingly depends on attentional and executive resources in later life.

Complex locomotor activities place greater demands on balance control, visuomotor coordination, and executive resources than unobstructed level walking. Environmental variability produces larger changes in dynamic stability and requires the adoption of compensatory strategies to maintain balance, particularly in older adults (Raffegeau et al., 2022). Balance is maintained by controlling the center of mass of the body relative to the base of support, i.e., the area defined by the position of the feet when in contact with the ground (Bruijn and van Dieën, 2018). Studies examining obstacle crossing have consistently shown larger alterations in gait behavior than are typically observed during level walking (Barolo et al., 2026; Plummer-D’Amato et al., 2012). These effects are compounded during cognitive-motor dual tasks. Older adults who perform cognitive tasks while negotiating obstacles exhibit reductions in walking speed, changes in step timing and foot placement, increased toe clearance, and decrements in cognitive-task performance, consistent with competition for shared attentional resources and adjustments to walking kinematics to reduce fall risk (Hamacher et al., 2019; Chen and Huang, 2008). In addition, a small reduction in path width with a constant cognitive load leads to larger dual-task costs in gait speed, stride length, and step width in older adults (Hennah et al., 2021), suggesting that increasing motor demands impaired both gait and cognitive performance despite no increase in cognitive-task difficulty.

Thus, increasing motor demands appear to substantially alter gait control even when cognitive-task requirements remain unchanged. These findings are consistent with dual-task theories that cognitive-motor interference emerges when concurrent tasks compete for limited attentional and executive-control resources (Wickens, 2008). As locomotor demands increase, a larger proportion of available resources must be devoted to maintaining gait stability and safe navigation, leaving fewer resources available for concurrent cognitive processing (Woollacott and Shumway-Cook, 2002). Studies manipulating motor demand demonstrate that dual-task interference cannot be attributed solely to increasing cognitive load. Instead, age-related dual-task costs appear to arise from competition for shared resources whenever either cognitive demands or motor-control demands exceed available capacity.

The present study addresses a major gap in the literature. Most studies have manipulated cognitive and motor demands separately, limiting our understanding of how these sources of demand jointly influence cognitive–motor performance. The present study addresses this issue by having participants perform cognitive and locomotor tasks simultaneously and then systematically varying difficulty within the same tasks. This manipulation is different from other studies that varied different motor task types (e.g., even surface vs. obstacle: Raffegeau et al., 2022) or different cognitive task types (e.g., simple RT, choice RT, discrimination: Bherer et al., 2005). Cognitive load was manipulated systematically within the same *n*-back task by increasing the number of items evaluated in working memory (Herff et al., 2014). Differences in performance between the n-back tasks primarily reflect added load on working memory and recruitment of additional executive control processes. The tasks require the individual to dynamically maintain, update, and manipulate information in active memory while continuously filtering out irrelevant stimuli. Influences such as processing speed, task strategies, motivation and fatigue are essentially held constant as load increases. By increasing working-memory requirements, we can examine how progressively greater cognitive demands affect gait performance and whether these effects differ with age. Previous studies have shown that younger and older adults exhibit increased prefrontal activation as cognitive load increased from lower-to higher-demand *n*-back tests during walking (Fraser et al., 2016). Older adults generally perform more poorly than younger adults, and the compensatory increases in cortical recruitment associated with aging appear to help maintain dual-task performance. Here, motor load, or difficulty, was manipulated by asking participants to walk on a treadmill at three, increasing inclines. Thus, our experiment systematically varied both working-memory load (0-back, 1-back, 2-back) and locomotor demand (level, intermediate, highest treadmill inclines).

To ensure simultaneous performance of both tasks, participants walked on a treadmill and stimuli were presented at a constant rate. Our design limits task switching as both tasks are continuous and cannot be stopped. Treadmill walking has been shown to improve cognitive performance during dual-task walking due to an increase in automaticity and a reduction in the activation of the prefrontal cortex (Baek et al., 2023; Penati et al., 2020), and in some cases, older adults were able to maintain similar levels of dual-task performance to younger adults (Baek et al., 2023). However, by adjusting the slope of the treadmill, participants must increase the height of the center of mass of the body with each step (Dewolf et al., 2021). Thus, uphill walking requires additional motor output to lower limb muscles (Franz and Kram, 2014) and increased sensorimotor integration and attentional processing compared to level walking (Bradford et al., 2016).

Our approach permits direct examination of the independent and interactive effects of increasing cognitive and motor demands on dual-task performance. We hypothesized that as cognition and motor load become more difficult, gait will be prioritized over cognitive performance. Specifically, participants will maintain stability as motor difficulty and cognitive load increase by increasing step width, relative stride length, or percent time in double support. Increasing step width and stride length should increase the participant’s base of support. Increasing the proportion of the gait cycle that both feet are on the ground, or percent double support, should also increase stability. However, if resources are unable to accommodate the load, as is likely in older adults, increased cognitive load and motor difficulty will lead to decreases in both cognitive performance and gait stability.

## Methods

### Participants

Sixty-seven individuals participated in this study. Younger adults (YA, *n* = 30, M = 19.87 years, SD = 1.25, range = 18–23 years; 13 female, 1 nonbinary; Education = 13–16 years) were recruited from the Claremont Colleges and received partial course credit in introductory psychology courses for their participation. Older adults (OA, *n* = 37, M = 72.63 years, SD = 6.41, range = 61–85 years; 22 female; Education = 12–20 years) were recruited from local walking and cycling groups in the Claremont, CA community; participants walked or cycled multiple times per week for at least an hour. They received financial compensation ($50/session) for their participation. Participants were excluded prior to participation if they reported a history of neurological disease or injury, stroke, cardiac arrest, loss of consciousness exceeding 2 min, did not have normal or corrected to normal hearing, or had mobility/walking impairments requiring mobility aids. Older adults also completed the Montreal Cognitive Assessment (MoCA; https://www.mocatest.org/) (M = 26.82, SD = 2.77, range = 18–30) to screen for cognitive impairment. In our sample, 82% of older adults scored at or above the recommended “unimpaired” cutoff score of 26. Although the remaining six participants scored between 18 and 25, suggesting possible mild cognitive impairment (Nasreddine et al., 2005), four reported that English was their second language which potentially affected the language portion of the MoCA. All participants provided informed consent approved by the Claremont McKenna College Institutional Review Board.

One young adult data set was excluded due to equipment failure. Four older adult data sets were excluded because the participants could not perform either the *n*-back or walking tasks successfully. Thus, a total of 62 participants were included in the reported analyses with 29 younger adults and 33 older adults. A power analysis using an approximation from the repeated-measures ANOVA framework to achieve 80% power for a three-way interaction of age, cognitive load and motor difficulty interaction indicated a minimum sample size of 20 participants per group (G*Power3, Faul et al., 2007). To be conservative, we recruited 25–35 participants per group as our target sample size.

### Apparatus: motion capture system

Kinematic data were collected using a Qualisys Motion Capture System consisting of 6 Qualisys Oqus and Miqus series cameras (Qualisys AB, Gottham, Sweden). The cameras were positioned to ensure comprehensive spatial coverage around the treadmill area (Figure 1, Left). Five cameras captured the entire marker set from the lowest possible marker (i.e., toe or heel) to the pelvis markers, and one camera focused on the feet. The experimental capture volume was calibrated prior to data collection at each incline using a Qualisys calibration wand (750.4 mm) until the residual error for all cameras was under 1 mm.

**Figure 1:**
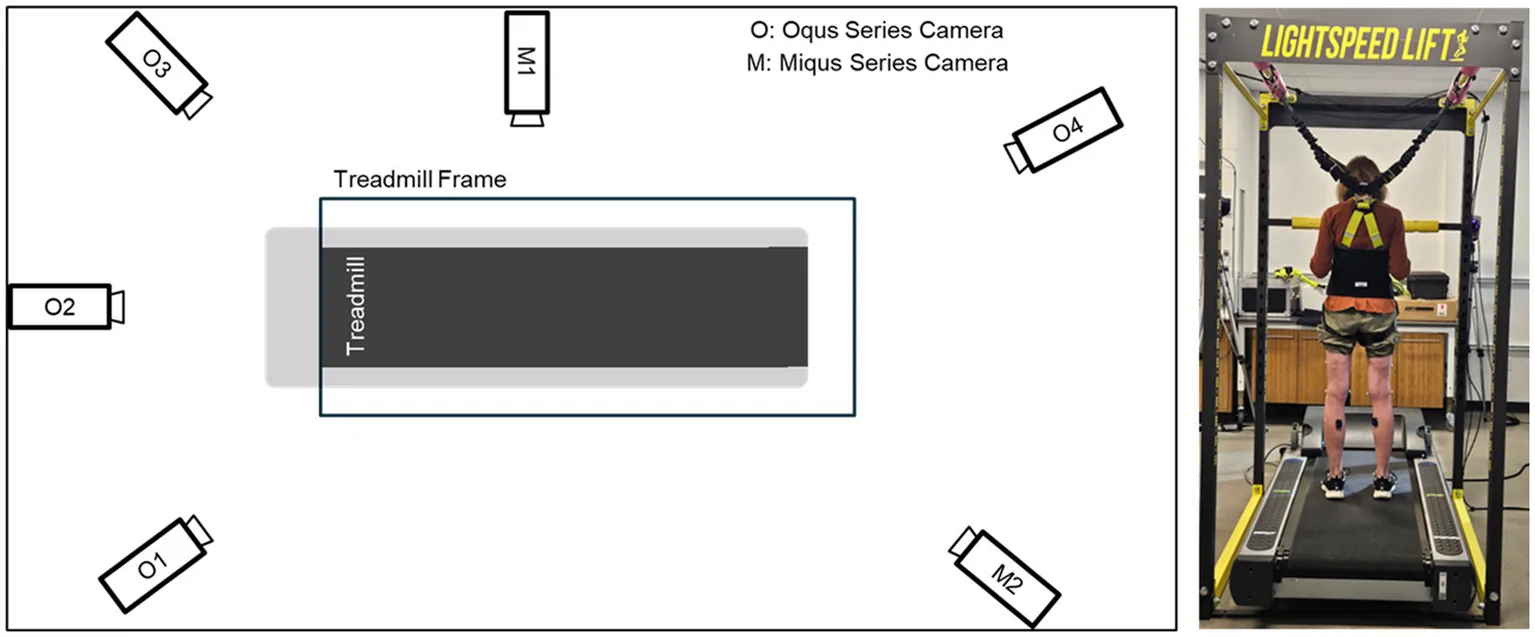
Left: schematic representation of Qualisys Oqus and Miqus cameras arrangement (one directly in front, 2 angled to the front and sides, 1 to the right side of the treadmill, and 2 angled at the back and sides of the treadmill). Right: treadmill with frame, front catch bar and harness.

Data were recorded and processed using Qualisys Track Manager (QTM) software (Version 2023.3) at a sampling frequency of 100 Hz. Retroreflective spherical markers of 9.5 mm in diameter (B&L Engineering) were placed on anatomical landmarks according to the Conventional Gait Model [HAS-Motion. (n.d.). *Visual3D documentation: Marker set guidelines*. Visual3D Wiki; Figure 2]. Specifically, critical markers to generate the model included three markers on the pelvis according to the Helen Hayes/ Newington model, two markers on each knee to determine the femur length (calculated as the distance from the hip to the knee joint) as well as the knee joint diameter, plus two markers on each ankle, and two at the toes of each foot. Tracking markers were placed on both thighs, knees, calves and toes. Kinematic data were synchronized using a Qualisys camera sync unit. Triggers were sent by E-prime 3.0 software (Psychology Software Tools, Pittsburgh, PA) to the recording at the initiation and termination of the cognitive task as well as the beginning of each trial.

**Figure 2:**
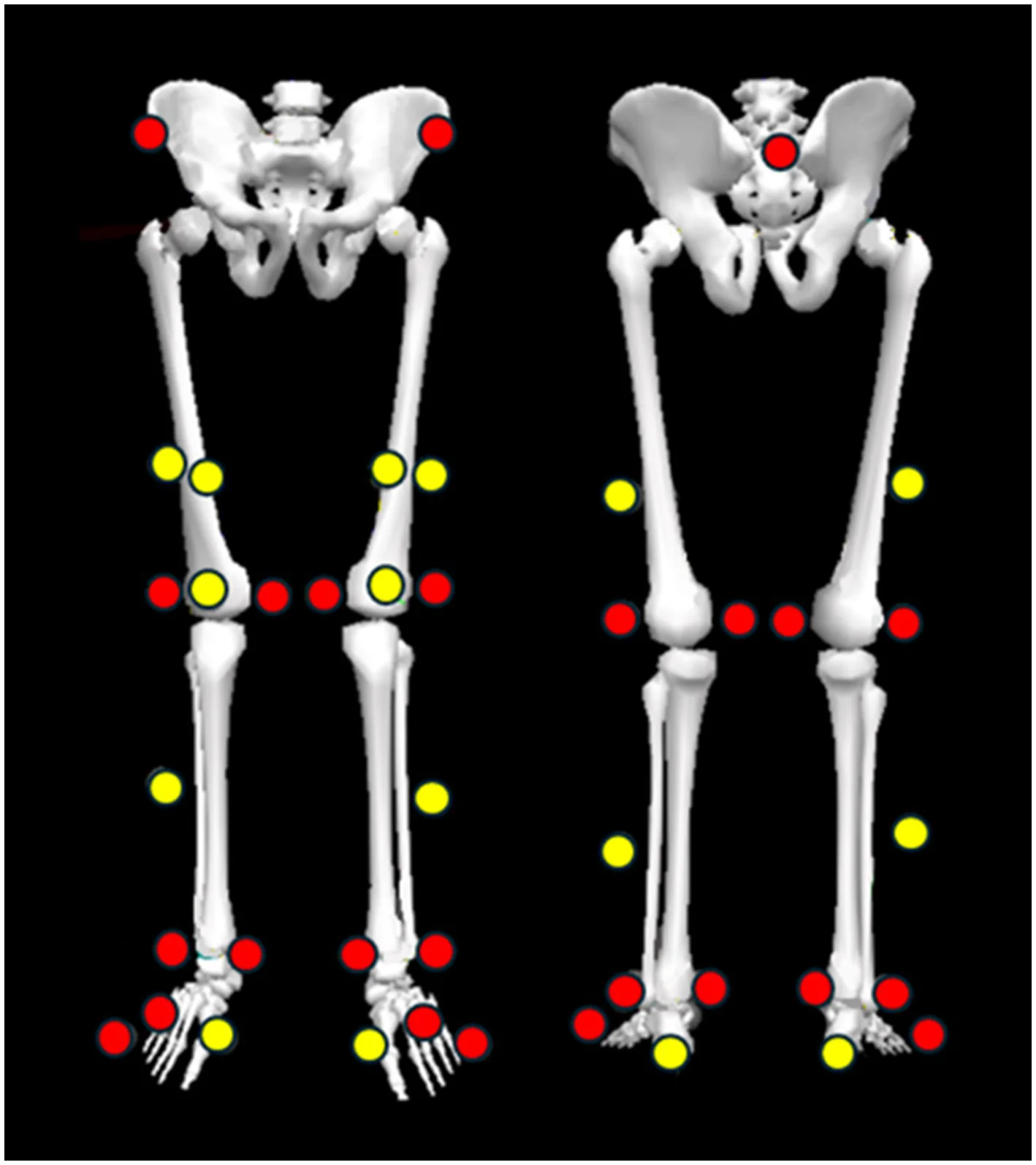
Front (left) and back (right) views of marker-placement diagram in Visual 3D (Version 2025.10.2; HAS-Motion, Waterloo, Ontario, Canada) for lower-extremity optical motion capture. Red dots indicate critical markers. Yellow dots indicate tracking markers.

### Procedure

Participants provided demographic information and completed the MoCA. To prevent falling, participants wore a full-body harness that was secured via a Lightspeed Lift 500 metal frame (lightspeedlift.com; Figure 1, Right). A handrail at the front of the treadmill was available for participants to regain balance if necessary. Additionally, an experimenter was positioned to the side of the treadmill to spot the older adult participants.

Prior to experimental trials, participants walked at all three inclines to familiarize them with the treadmill. At each of the inclines, the speed of the treadmill was adjusted to a participant’s preferred walking speed. Participants selected a single preferred walking speed that they could comfortably sustain across all three incline conditions, including the highest one. This approach ensured that any observed differences in gait strategy across inclines reflected kinematic adaptation to increasing biomechanical demands rather than artifacts of a pace that was mismatched to individual capability. This is an important consideration when comparing age groups that differ in physical capacity and gait adaptability. It also helped equate perceived motoric effort across participants, although perceived effort was not experimentally measured. The preferred speed at the highest incline was used for all experimental trials. For a subset of participants (*n* = 26), kinematic data was collected for a no-task walking condition at all inclines.

During the experiment, participants walked at their preferred speed on a treadmill. Each participant performed the auditory *n*-back working memory task at three levels of cognitive load—0-back, 1-back, and 2-back—while simultaneously walking at each of three levels of motor difficulty—level (0°), intermediate (2.3°) and highest (4.6°) inclines. For the *n*-back tasks, participants listened to sequences of spoken letters (played through headphones) presented one at a time (Figure 3). Auditory letter stimuli were spoken by an adult male speaker and were presented over Gtheos 2.4GHz Wireless Gaming Headphones. Key-press responses were made on a Logitech M185 wireless mouse. For all *n*-back tasks, participants wore earphones and held a wireless mouse in their dominant hand; they were told to press the left button every time they heard a target letter, and to press the right button for all non-target letters. For the 0-back task, the target was a pre-designated letter (e.g., the letter “X”), while non-targets were all other letters. This required basic attention and auditory processing but minimal working memory. The 0-back task served as baseline control. Note, the 0-back condition is a minimally challenging cognitive task that allows consistency as to the cognitive processing being performed while walking, unlike a walking condition with no cognitive task in which there is no control as to the cognitive processes participants are performing. For the 1**-**back task, participants indicated a target when the currently heard letter was a match to the letter heard *one trial earlier* and a non-target when the currently heard letter did not match the one heard previously. For the 2-back task, which represented a higher-load working memory challenge, participants indicated a target when the current auditory letter matched the one heard *two trials earlier* and indicated a non-target when the letters did not match.

**Figure 3:**
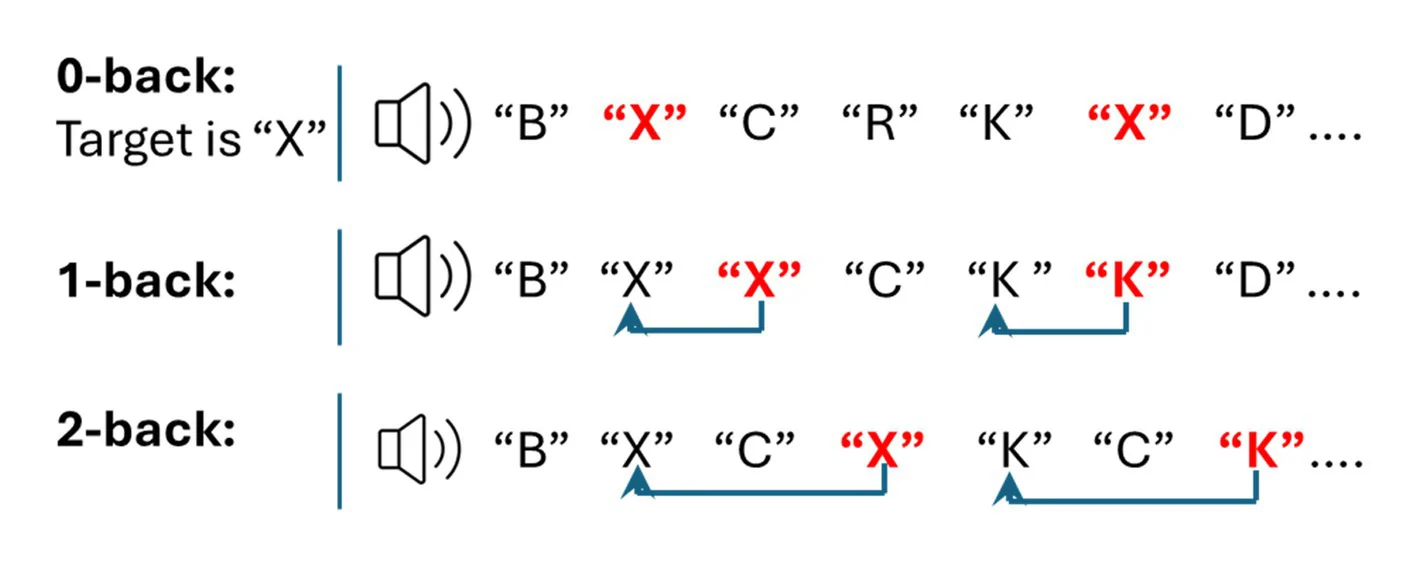
Auditory 0-back, 1-back, and 2-back tasks. Separate buttons for target (bold red) and non-target letters (black). 0-back task: Target is specified. 1-back task: target matches previous letter. 2-back task: target matches letter presented two previously.

For each task, letter stimuli were presented for a duration of 500 ms separated by an inter-stimulus interval (ISI) of 3,000 ms. Each task had 30 trials in which target letters comprised 20% of the trials (6 trials) and non-target letters made up 80% (24 trials). Stimuli were presented in a pseudorandomized order, with the constraint that no identical stimulus type could be repeated more than three times in a row. Participants were given 10 practice trials prior to each task session as they walked at their preferred speed for the given cognitive task and incline condition; each session was approximately 3 min in duration. Accuracy and response times (RTs) were recorded.

All participants began with the 0-back task at the level incline to acclimate them to the dual-task paradigm. Following the level incline, incline order and task order were counterbalanced (Supplement A); all three *n*-back tasks were performed at each incline before changing incline. In total, each participant completed 9 tasks (3 *n*-back tasks × 3 inclines). Participants took breaks between each task. The motion capture system was recalibrated after each change of incline. The entire testing session was approximately 2 h in duration.

### Kinematic data processing

For each participant, during the experimental session a 10 s static recording was used to ensure marker visibility. All markers were labeled, and an AIM (automatic identification of markers) model was generated to be applied to the subsequent kinematic recordings. The recordings were then examined to correct any mislabeled markers and ensure successful tracking for the duration of the task. A session was re-recorded if model-generating markers fell off for more than 10% of the task. Successful tracking for all model-generating markers was 98–100%. Gap-filling settings were set to fill a maximum of a 10-frame (100 ms) gap, defaulting to a polynomial interpolation. Gap fill was primarily applicable for ensuring successful tracking of the medial knee markers, particularly when the markers were obscured by the other leg. Qualisys files were then exported to C3D for further processing in Visual 3D (Version 2025.10.2; HAS-Motion, Waterloo, Ontario, Canada). The Conventional Gait Model was applied to each data set. For each dynamic trial, one heel strike (HS) and toe off (TO) event for each foot were added manually. Additional HS and TO events were generated using pattern-based event creation target pattern recognition (TPR) commands, with a TPR window of 4 frames. Tolerance was adjusted separately for older and younger adults to account for age-related variation in TPR success. Missing events were labeled and fixed manually whenever possible.

Steps were defined from the HS of one foot to the HS of the other foot. Strides were defined as the cycle from HS of one foot to the next HS of the same foot. Step width, toe clearance, stride frequency, relative stride length and percent double support kinematic measures from HS and TO events. Missing events were excluded from the calculation of the variables resulting in the typical removal of less than 1% of the data, and no more than 6% data removal for those with tracking issues. For each participant, steps were lost due to lost markers. No participant lost more than 6% of their data. Nine datasets had 1–4 missing strides in 1–3 tasks due to heel marker obstruction during recording. One additional dataset had marker tracking issues such that the heel marker became obstructed for multiple strides on three of the tasks, resulting in the loss of between 7 and 17 strides. Thus, there was a range of 68–121 strides contributing to each participant’s averaged stride variables in each task.

*Step width (m)* was calculated as the vector distance between the heel marker of one foot at HS to the heel marker of the other foot at HS along the y-axis (mediolateral axis).

*Toe clearance (m)* was defined as the minimum z-value (height) of the toe marker from the treadmill during the swing phase of that foot.

*Stride frequency (Hz)* was calculated as the number of strides per second, or the inverse of stride time where stride time is the time from HS to the next HS of the same foot.

*Relative stride length* was calculated as stride length divided by leg length. Stride length was calculated as the vector distance (m) between heel marker of one foot at HS to the heel marker of the same foot at the next HS. Leg length was measured from the hip marker to the heel of one leg.

*Percent double support* was calculated as the percent of time that both feet were on the ground (stance) relative to the total stride time, or the time between the HS of one foot to the TO of the other foot.

### Behavioral data processing

For each participant and condition, cognitive task performance was measured using *d*-prime (*d*′) and response time (RT) measures. *D*′ is a performance metric that takes into account participants ability to distinguish the true target from other non-target stimuli as well as their personal decision biases. It mathematically separates a person’s underlying perceptual sensitivity from their willingness to guess, which can artificially inflate accuracy. In the context of the *n*-back task, *d*′ provides an index of working memory performance that reflects the executive processes required to maintain, update, and discriminate task-relevant information (Haatveit et al., 2010; Stanley et al., 2015). Hits occur if a target is perceived when a target is presented. False alarms occur if a target is perceived but no target is presented. *D*′ was calculated as *z*(hit rate) − *z*(false alarm rate), with correction for hit and false alarm rates of 0 or 1 to eliminate infinite *z* values (Macmillan and Creelman, 1991). A *d*′ score of 0 indicates an inability to distinguish signal from noise (i.e., random guessing or chance). Higher *d*′ scores indicate better discrimination between targets and non-targets or greater cognitive sensitivity. Mean response times (RTs) for correct trials were also calculated. Individual responses faster than 150 ms were excluded from the means as anticipation errors (less than 1% of trials).

### Statistical modeling and analyses

Statistical analyses were conducted in SPSS (v. 24) for behavioral performance (*d*′ and mean RT) on the *n*-back task and to compare the task vs. no task baseline conditions across kinematic variables. A mixed-model analysis of variance (ANOVA) was carried out with the between-subjects factor of age group (2: younger, older adults) and within-subject factors of cognitive load (3: 0-back, 1-back, 2-back) and motor difficulty (3: level, intermediate, and highest incline). All main effects and interactions are reported with Greenhouse–Geisser and Bonferroni corrections. *Post-hoc t*-tests controlled for multiple comparisons using Bonferroni corrections.

Statistical analyses of kinematic variables were conducted in RStudio (2024.12.0.467) using the lme4 (Bates et al., 2015) and lmerTest (Kuznetsova et al., 2017) packages. Because preferred speed is strongly related to spatiotemporal gait parameters (e.g., stride length and stride frequency are direct functions of speed), it was included as a covariate in all statistical models. This served two purposes. First, it accounted for individual differences in gait parameters attributable to differences in preferred speed rather than age, motor difficulty, or cognitive load. Second, it allowed us to examine whether age-related or incline-related effects on gait persisted independently of speed. This approach allowed us to distinguish age-related differences in gait that were explained by differences in preferred speed from those that remained independent of speed.

Each of the five gait variables (relative stride length, stride frequency, percent double support, step width, toe clearance) was modeled using linear mixed-effects models with subject-level random effects to account for the repeated-measures structure (nine observations per participant). To determine which model to use, variable outcomes were initially analyzed using a base model with following fixed effects: Age Group (2: young, old), Motor Difficulty (3 inclines: level, intermediate, highest inclines), Cognitive Load (3 *n*-back loads: 0-back, 1-back, 2-back), and preferred walking speed. A fully crossed three-way interaction (Age Group × Motor Difficulty × Cognitive Load) was tested against a simpler additive structure (Age Group × Motor Difficulty + Cognitive Load + Preferred Speed) for each outcome via a likelihood ratio test. In no case did the three-way interaction significantly improve fit (all *ps* > 0.05), therefore the additive structure was retained throughout as the fixed-effects base model. Because participants may vary in how their gait adapts across incline, a model including random slopes for incline was compared against a random-intercept-only model for each variable. Random slopes significantly improved fit for relative stride length [*χ*^2^(5) = 161.15, *p* < 0.001], stride frequency [*χ*^2^(5) = 124.60, *p* < 0.001], double support time [*χ*^2^(5) = 45.65, *p* < 0.001], and toe clearance [*χ*^2^(5) = 824.72, *p* < 0.001]. The random-intercept-only model was retained for step width because including random slopes in the model showed a singular fit with near-zero variance suggesting overfitting.

RT and *d*′ were added to the base model as cognitive predictors of gait. Each was tested for a main effect and their interactions with Age Group, Motor Difficulty, and Cognitive Load. Where more than one interaction was individually significant, a joint model including all significant interaction terms was fit to evaluate whether effects were independent or attributable to shared variance. Pairwise comparisons of estimated marginal means and estimated marginal slopes (for continuous-by-categorical interactions) were conducted using the emmeans package with Kenward-Roger degrees of freedom adjusted for multiple comparisons using the Tukey method when necessary (Lenth and Piaskowski, 2026; Brysbaert and Debeer, 2025).

Given the number of gait variables, cognitive measures, and model terms examined, no formal correction for multiple comparisons was applied during initial model selection across the full set of models. This reflects a hypothesis-generating framework, rather than strictly confirmatory analytic framework, in which each gait variable and cognitive predictor was evaluated as a distinct question. To mitigate the risk of false-positive findings under this approach, several converging criteria were required before an interaction was interpreted as reliable: statistical significance of the likelihood ratio test, significance of at least one individual coefficient (rather than a diffuse effect spread across non-significant terms), and, where feasible, agreement between AIC and BIC model comparison criteria. Findings meeting only some of these criteria were explicitly downgraded and reported as “exploratory,” indicating they may require additional exploration.

## Results

### Cognitive performance on the *n*-back task

*D′* An Age Group (2: OA, YA) by Cognitive Load (3: 0-back, 1-back, 2-back) by Motor Difficulty (3: level, intermediate, highest) mixed-model ANOVA was conducted for *d*′ scores (Figure 4, left panel). Younger adults demonstrated significantly greater discrimination sensitivity than older adults, *F*(1, 59) = 12.14, *p* < 0.001, *η_p_*^2^ = 0.17, with mean *d*′ scores of 3.13 (SE = 0.06) for younger adults compared with 2.86 (SE = 0.05) for older adults. Cognitive Load also significantly affected discrimination sensitivity, *F*(1.89, 111.39) = 33.19, *p* < 0.001, *η_p_*^2^ = 0.36, indicating greater *d*′ for the 0-back condition than for both the 1-back and 2-back conditions. This is consistent with the interpretation that the 0-back task imposed the lowest cognitive demands. Moreover, this effect differed across age groups, as reflected by a significant Age Group × Cognitive Load interaction, *F*(1.89, 111.39) = 10.62, *p* < 0.001, *η_p_*^2^ = 0.15. Follow-up comparisons indicated that older adults exhibited an overall 24% reduction in *d*′ with significant reductions in d′ with increasing cognitive load (*p*s < 0.02). Younger adults demonstrated a 5.1% change in d′ with a significant decline only between the 0-back and 1-back conditions (*p*s < 0.001). The significant main effect of Motor Difficulty, *F*(1.80, 105.99) = 15.21, *p* < 0.001, *η_p_*^2^ = 0.21, depended on Cognitive Load, as indicated by a significant Cognitive Load × Motor Difficulty interaction, *F*(3.27, 193.04) = 12.11, *p* < 0.001, *η_p_*^2^ = 0.17. Collapsing over age group, at the level incline, there was a 25.6% decrease in d′ from lowest to highest cognitive load; *d*′ for the 0-back task was greater than for the 1-back and 2-back tasks (both *ps* < 0.001). At the intermediate incline, there was an 8.7% decline in *d*′; *d*′ for the 0-back task exceeded that of the 2-back task (*p* < 0.01). At the highest incline, there was a 10.2% decline in *d*′; *d*′ for the 0-back task was higher than for both the 1-back and 2-back tasks (both *p*s < 0.001), whereas the 1-back and 2-back conditions did not differ significantly. There was no 3-way interaction with Age Group (*p* < 0.99). All remaining interactions were not significant. In addition, there was no strong indication of strong speed-accuracy tradeoffs; *d*′ scores were not significantly correlated with corresponding condition mean RTs (all *p*s > 0.10), with the exception of the level incline 2-back condition (*r* = −0.33, *p* = 0.011).

**Figure 4:**
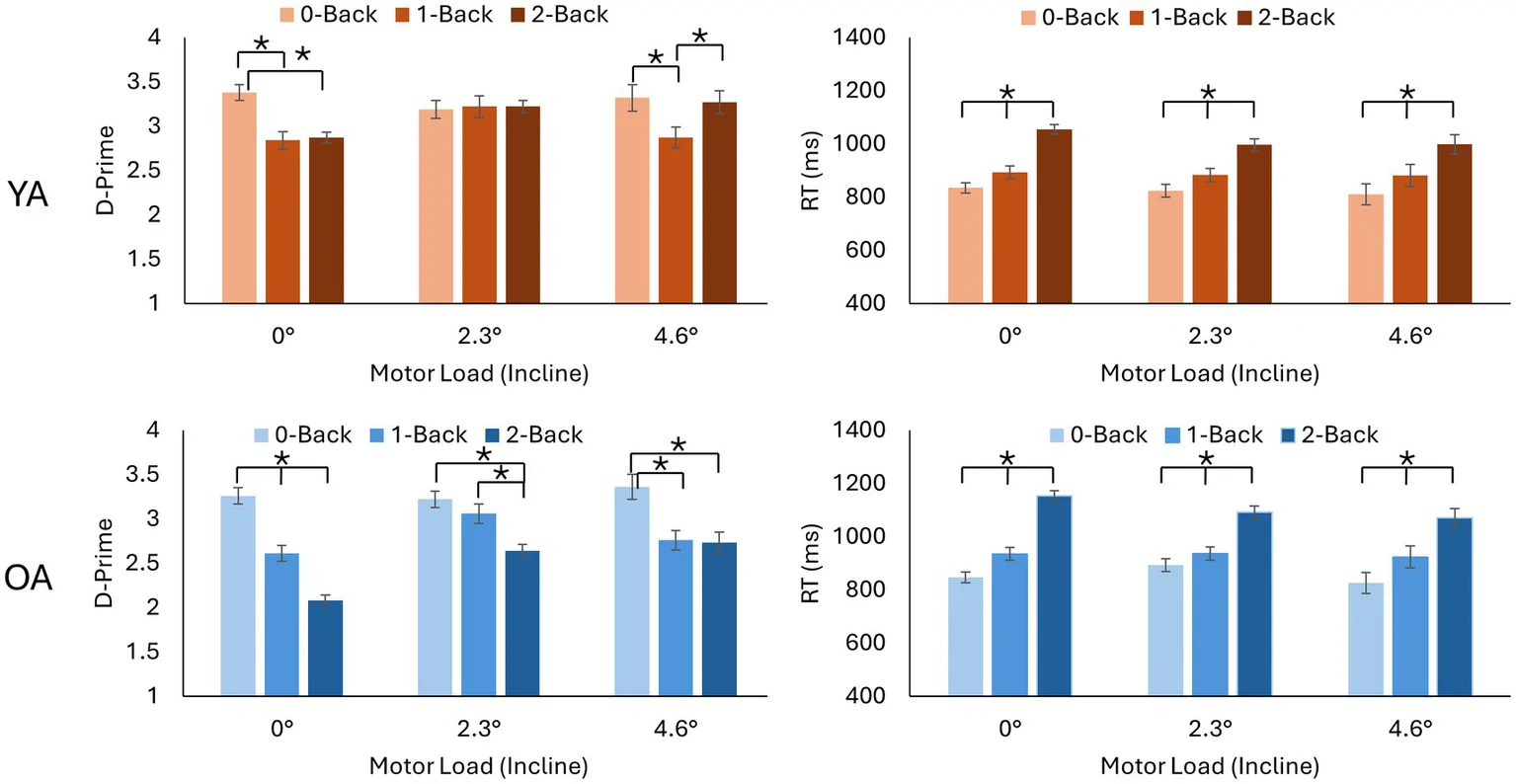
Left: Cognitive performance (*d*′) on auditory *n*-back tasks by motor load (or difficulty) and age group (young adults (YA) are in red; older adults (OA) are in blue). Error bars represent SE. Right: Mean response time data for correct responses (*RTs*) by age, *n*-back task, and motor load (or difficulty). Error bars represent SE. Brackets and stars indicate significant pairwise comparisons (*p* < 0.05). Increased cognitive load decreases *d*′ and increases *RTs* for both age groups.

*RTs*. Younger adults responded significantly faster than older adults, *F*(1, 59) = 5.13, *p* < 0.03, *η_p_*^2^ = 0.08, with mean RTs of 874.20 ms (*SE* = 21.44) compared with 945.77 ms (SE = 20.10) for older adults (Figure 4, right panel). A robust effect of Cognitive Load, *F*(1.22, 71.82) = 88.50, *p* < 0.001, *η_p_^2^* = 0.60, showed that RTs increased significantly from the 0-back to the 1-back and from the 1-back to the 2-back condition (all *p*s < 0.001), supporting the effectiveness of the cognitive load manipulation. A significant main effect of Motor Difficulty was also observed, *F*(1.87, 110.48) = 5.97, *p* < 0.004, *η_p_*^2^ = 0.09, indicating faster responses at higher treadmill inclines, with RTs significantly faster at the highest compared with the level incline (RT difference = 17.11 ms). The significant Cognitive Load × Motor Difficulty interaction, *F*(2.84, 167.76) = 4.76, *p* < 0.004, *η_p_*^2^ = 0.08, further indicated that RTs increased with cognitive load at every incline (all *p*s < 0.001) with a 17.5% RT difference between the lowest load (0-back, level incline) and highest load (2-back, highest incline) conditions. Follow-up analyses indicated that for both age groups, each increase in cognitive load produced a significant increase in RT at each incline (all *ps* < 0.002). All remaining main effects and interactions were nonsignificant.

In summary, parametric increases in cognitive load and motor difficulty (i.e., incline) had a systematic influence on behavioral performance. Working memory performance was affected by age to a greater extent than response speed.

### Kinematic performance

#### 0-back vs no task comparisons

To determine whether the 0-back condition provided an appropriate low cognitive load baseline, we compared the no Back (walking only) and 0-back conditions separately, in a subgroup of participants (*N*_YA_ = 7, *N*_OA_ = 17), for each age group and incline using Age Group (2: older, younger) × Baseline (2: no back, 0-back) × Motor Difficulty (3: level, intermediate, highest) mixed-model ANOVAs with preferred walking speed entered as a covariate for step width, toe clearance, stride frequency, relative stride length, and percent double support. For all kinematic variables, the 0-back condition did not differ significantly from the no back walking condition for either age group (all main effects and interaction *p*s > 0.12). These findings indicate that the 0-back condition imposed minimal additional cognitive demands and therefore served as an appropriate behavioral baseline for the subsequent dual-task analyses.

### Kinematic variables

*Step width* decreased significantly with Motor Difficulty, *F*(2, 495) = 5.75, *p* = 0.003, *η_p_*^2^ = 0.02, 95% CI [0.0, 0.05], specifically between the level (M = 0.125 m, SE = 0.003) and intermediate (M = 0.121 m, SE = 0.003) inclines. In addition, there was a significant effect of d′, *F*(1, 503) = 5.17, *p* = 0.023, *η_p_*^2^ = 0.01, 95% CI [0.0, 0.03], though this effect did not survive BH correction (see Supplement B). However, there was a significant *d*′ × Motor Difficulty interaction, *F*(1, 495) = 5.41, *p* = 0.005, *η_p_*^2^ = 0.02, 95% CI [0.0, 0.05], BH-adjusted *p* = 0.014; *χ*^2^(2) = 10.87, *p* = 0.004 which remained robust under correction. Higher *d*′ predicted greater step width specifically at the highest incline [*emtrends*: *β* = 0.008, 95% CI (0.004, 0.012)], but no relationship was present on level ground [*β* = 0.001, 95% CI (−0.002, 0.004)] or at the intermediate incline [*β* = −0.001, 95% CI (−0.004, 0.003)]. There were no other significant main effects or interactions.

*Toe clearance* differed significantly as a function of Age Group, *F*(1, 60) = 23.31, *p* < 0.001, *η_p_*^2^ = 0.28, 95% CI [0.11, 0.44]. Younger adults exhibited greater toe clearance at every level of incline (M = 0.67 m, SE = 0.002) compared to older adults (M = 0.06 m, SE = 0.002). There were significant effects of *d*′, *F*(1, 393.6) = 6.55, *p* = 0.011, *η*^2^ = 0.02, 95% CI [0.0, 0.05], and a *d*′ × Motor Difficulty interaction, *F*(2, 399.6) = 8.59, *p* < 0.001, *η*^2^ = 0.04, 95% CI [0.01, 0.08], with a positive relationship between *d*′ and toe clearance specific to level-ground walking [*emtrends*: level: *β* = 0.002, 95% CI (0.0009, 0.0021)], intermediate: [*β* = −0.00001, 95% CI (−0.0007, 0.0005)], highest inclines: [*β* = −0.0001, 95% CI (−0.0006, 0.0008)]. All remaining main effects and interactions were nonsignificant.

*Stride frequency* was influenced by both Age Group and Motor Difficulty (Figure 5A). There also was a significant effect of the Speed covariate, *F*(1, 60) = 6.21, *p* = 0.015, *η_p_^2^* = 0.1, 95% CI [0.0, 0.25]. Older adults demonstrated significantly greater stride frequency than younger adults, *F*(1,60) = 19.36, *p* < 0.001, *η_p_*^2^ = 0.24, 95% CI [0.08, 0.41], with mean stride frequencies of 0.91 Hz (SE = 0.01) for older adults and 0.83 Hz (SE = 0.01) for younger adults. A significant effect of Motor Difficulty, *F*(2, 62) = 48.04, *p* < 0.001, *η_p_*^2^ = 0.61, 95% CI [0.45, 0.71], indicated that stride frequency generally decreased with increasing incline. However, this effect differed across age groups, as reflected by a significant Age Group × Motor Difficulty interaction, *F*(2, 62) = 6.63, *p* < 0.002, *η_p_*^2^ = 0.18, 95% CI [0.03, 0.33]. Younger adults displayed progressive decreases in stride frequency with each increase in incline, whereas older adults reduced stride frequency only between the level and intermediate inclines, with no additional adjustment at the highest incline. All remaining main effects and interactions were not significant (all *p* > 0.06). Cognitive performance also significantly affected stride frequency. There was a significant effect of RT, [*F*(1, 415) = 5.34, *p* = 0.02, *η_p_*^2^ = 0.01, 95% CI (0.0, 0.04); *χ*^2^(1) = 8.79, *p* = 0.003], and a three-way Age Group x Motor Difficulty × RT interaction, *F*(2, 413) = 5.07, *p* = 0.006, *η_p_*^2^ = 0.02, 95% CI [0.0, 0.06]; *χ*^2^(5) = 18.47, *p* = 0.002. There was a negative relationship between RT and stride frequency specific to older adults at the level incline only [*β* = −0.0069, 95% CI (−0.0101, −0.0037)]; no other Age Group × Motor Difficulty comparison showed this relationship. There were significant *d*′ × Motor Difficulty, *χ*^2^(2) = 9.96, *p* = 0.007, *η_p_*^2^ = 0.02, 95% CI [0.0, 0.04], and *d*′ × Cognitive Load, *χ*^2^(2) = 8.58, *p* = 0.014, *η*^2^ = 0.01, 95% CI [0.0, 0.04], interactions. Higher *d*′ predicted lower stride frequency at the intermediate incline and higher stride frequency under the highest load (2-back) condition. A three-way Age Group × Motor Difficulty × *d*′ interaction was tested and rejected on the omnibus/coefficient mismatch criterion, though one specific cell (intermediate incline × hardest *n*-back) showed a strong, individually significant three-way effect (*β* = −0.024, *p* < 0.001) which may warrant follow-up.

**Figure 5:**
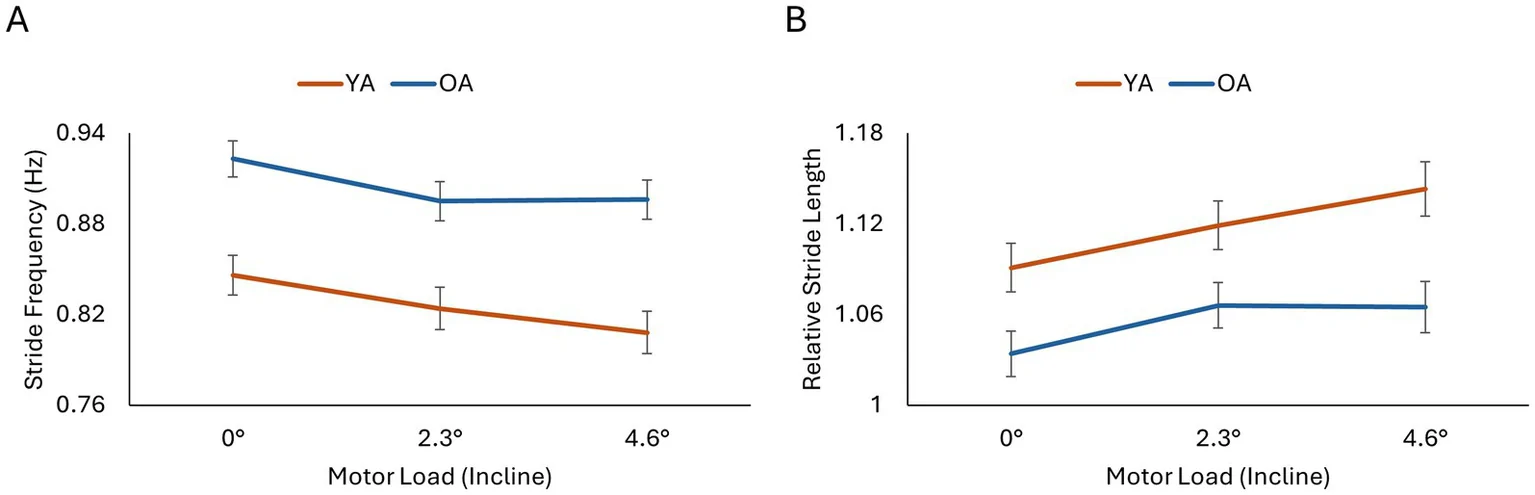
**(A)** Age group × motor difficulty interaction for stride frequency (strides/s). **(B)** Age group × motor load interaction for relative stride length (stride length (m)/leg length (m)). For young adults, stride frequency decreases and relative stride length increases with incline, but for older adults, they did not change for the highest incline.

*Relative stride length* was influenced by both Age Group and Motor Difficulty (Figure 5B). The Speed covariate was significant, *F*(1, 60) = 199.99, *p* < 0.001, *η_p_^2^* = 0.77, 95% CI [0.67, 0.83], indicating that a longer stride length is associated with faster preferred speeds. After controlling for preferred walking speed, older adults exhibited significantly shorter relative stride lengths than younger adults, *F*(1, 61) = 7.38, *p* < 0.009, *η_p_*^2^ = 0.11, 95% CI [0.01, 0.27], with mean relative stride lengths of 1.06 (SE = 0.02) for older adults compared with 1.12 (SE = 0.02) for younger adults. Notably, the effect of Motor Difficulty differed across age groups, as reflected by a significant Age Group × Motor Difficulty interaction, *F*(2, 61) = 7.98, *p* < 0.001, *η_p_*^2^ = 0.22, 95% CI [0.04, 0.36]. Younger adults progressively increased relative stride length with each increase in incline (all pairwise comparisons: *p* < 0.0001), whereas older adults increased stride length only between level ground and intermediate inclines, exhibiting little additional adjustment at the highest incline (*p* = 0.998). No other main effects or interactions reached significance (all *ps* > 0.07). Cognitive performance (RT and *d*′) also significantly affected relative stride length. RT showed a general effect in which slower RT was associated with longer relative stride length, *F*(1,412) = 8.34, *p* < 0.004, *η_p_*^2^ = 0.02, 95% CI [0.0, 0.06]; *χ*^2^(1) = 10.74, *p* = 0.001. An exploratory Age Group × Motor Difficulty × RT interaction favored by AIC but not BIC criterion, *χ*^2^(5) = 14.29, *p* = 0.014, indicated a positive relationship between RT and relative stride length at ground level for both age groups [*emtrends*: older: *β* = 0.0068, 95% CI (0.0032, 0.0104); younger: *β* = 0.0051, 95% CI (0.0002, 0.0101)], and at the intermediate incline for younger adults only [*β* = 0.0058, 95% CI (0.0010, 0.0106)]. This relationship was not present for older adults at the intermediate incline [*β* = −0.0030, 95% CI (−0.0077, 0.0016)], nor for either age group at the highest incline. This pattern suggests the relationship between RT and relative stride length is attenuated, specifically for older adults walking on an incline. *D*′ showed no main effect, but a significant *d*′ × Motor Difficulty interaction, *F*(2, 431) = 5.52, *p* = 0.004, *η_p_*^2^ = 0.02, 95% CI [0.0, 0.06]; *χ*^2^(2) = 10.86, *p* = 0.004, in which there is a negative relationship between *d*′ and relative stride length at ground level that is not present when walking on an incline.

*Percent double support* differed significantly as a function of age. Older adults spent a greater percentage of the gait cycle in double support than younger adults, *F*(1, 61) = 10.02, *p* < 0.002, *η_p_*^2^ = 0.14, 95% CI [0.02, 0.30], with mean values of 37.13% (SE = 0.62) for older adults compared with 34.46% (SE = 0.66) for younger adults. Percent double support increased with Motor Difficulty, *F*(1, 61) = 12.47, *p* < 0.001, *η_p_*^2^ = 0.29, and decreased with preferred Speed, *F*(1, 61) = 22.23 *p* < 0.001, *η_p_*^2^ = 0.27, 95% CI [0.10, 0.44]. Neither RT nor *d*′ showed any relationship with percent double support (RT: *χ*^2^(1) = 1.06, *p* = 0.302, *d*′: *χ*^2^(1) = 2.96, *p* = 0.085). All remaining main effects and interactions were nonsignificant.

### False discovery rate correction

We applied Benjamini-Hochberg (BH) correction for false discovery rate to all principal effects reported in the study (Supplement B). Both across the full set of tests and separately within each of the five gait outcomes. Of the significant effects, nearly all remained significant after correction. Only two findings did not survive correction: (1) the Age Group × Motor Difficulty × RT interaction for relative stride length (raw *p* = 0.034, BH-adjusted *p* = 0.071–0.083), which was already identified as exploratory under our pre-specified criteria; and (2) the main effect of *d*′ on step width (raw *p* = 0.023, BH-adjusted *p* = 0.047–0.053), which was superseded by the more specific *d*′ × Motor Difficulty interaction (BH-adjusted *p* = 0.014). This convergence between our *a priori* qualitative safeguards and formal FDR corrections provided additional confidence that the confirmed findings reported in this study were not attributable to chance across the multiple outcomes and models examined. Full uncorrected and FDR-adjusted *p*-values for all principal effects are provided in Supplement B.

## Discussion

The present study examined how increasing cognitive and motor demands jointly influence gait adaptation in healthy younger and older adults. In this dual-task study, participants performed a cognitive task designed to increasingly challenge executive function, or working memory, while concurrently walking on a treadmill at increasing motor difficulty, operationalized as increased incline. We hypothesized that progressively increasing executive demands during walking would interfere with the kinematic adaptations necessary to maintain stability, particularly in older adults who may experience age-related declines in executive function and physical adaptation capabilities (e.g., Lee and Lee, 2023). By systematically manipulating both working memory load (0-, 1-, and 2-back tasks) and biomechanical difficulty (level, intermediate, and highest treadmill inclines), the present study provides a detailed examination of how cognitive and locomotor systems share resources during increasingly demanding dual-task performance and whether age affects any gait adaptations that may occur.

Several findings emerged. First, our parametric manipulations successfully increased cognitive and motor demands across age groups. Second, contrary to the prediction that increasing executive demands would broadly disrupt locomotor control, we observed minimal changes in gait with increased cognitive load. Third, discrimination sensitivity (*d*′) and cognitive processing speed (RT) have distinctive effects on gait kinematics that are different from pure age-related effects.

### Cognitive performance confirms age-related executive resource limitations

The behavioral findings are consistent with prior studies demonstrating age-related reductions in working memory and executive control (Kirova et al., 2015). *D*′ decreased and RTs increased as *n*-back difficulty increased, with older adults exhibiting larger reductions in working memory performance than younger adults. Older adults had a 24% reduction in *d*′ between the lowest and highest cognitive and motor demand conditions while younger adults had only a 5% reduction and adapted to cognitive load manipulations after the first presentation of the tasks. Such age-related reductions in processing resources associated with aging have been associated with declines in inhibitory control as well as structural and functional changes within prefrontal cortex (Craik and Byrd, 1982; Hasher and Zacks, 1988; Park and Reuter-Lorenz, 2009).

Further, although motor difficulty (i.e., increasing inclines) exerted only modest effects on d′, combined cognitive and motor loads had a significant effect on response times, regardless of age. Between the lowest (0-back, level incline) and highest combined load (2-back, highest incline) conditions, RTs increased 17.5%. These findings support multiple-resource models suggesting that walking and executive function draw upon partially overlapping attentional resources, with performance decrements as combined demands require more of the available capacity (Wickens, 2008).

### Older adults exhibit limits to successful gait adaptation

Further, we confirmed known interactions of age and incline with several kinematic variables. Younger adults showed expected biomechanical changes to incline (Kimel-Naor et al., 2017), including decreased stride frequency and increased relative stride length while walking on the treadmill with relatively moderate increases in incline. Similar to other studies (e.g., Boyer et al., 2017), older adults exhibited lower toe clearance, slower preferred walking speeds, greater stride frequency, shortened relative stride length, and more time in double support during level walking. Although they adjusted to the first increase in incline similarly to younger adults, they failed to adapt as motor demands (inclines) increased. Both stride length and stride frequency appeared to reach a plateau despite an additional increase in incline. Uphill walking is particularly challenging for older adults due to reduced leg power (Franz and Kram, 2014) and age-related decline in sensorimotor integration (Bradford et al., 2016). Aging may reduce the flexibility or dynamic range of gait adaptation rather than prevent compensation altogether. Such an interpretation aligns closely with current theories emphasizing reduced physiological reserve rather than outright impairment in healthy aging (Seidler et al., 2010). Nonetheless, the age- and incline-related effects should be interpreted as effects independent of individual differences in preferred walking speed, rather than as representing the complete pattern of age-related locomotor adaptation.

One unexpected finding was the relative absence of large cognitive-load effects on gait kinematics. Of the kinematic variables, the only effect of cognitive load was on toe clearance: toe clearance decreased as cognitive load increased, and this occurred for both young and older adults. One extrapolation of this finding might be that there is a greater chance of tripping as cognitive load increases, but that this is not exaggerated in older adults. Importantly, it does not appear that cognitive load and motor difficulty potentiate one another, in either age group. Traditional dual-task theories would predict that increasing executive demands should impair the ability to generate adaptive gait adjustments, particularly in older adults (Woollacott and Shumway-Cook, 2002). However, increasing cognitive load alone produced relatively few changes across the primary kinematic variables, suggesting that healthy adults can maintain gait control even with increasing executive demands. This pattern, in part, may be a function of kinematic constraints when walking on a treadmill at a constant treadmill speed that is within the physical capability of the participants. Walking may be sufficiently automated that additional moderate executive demands do not substantially interfere with its control (Baek et al., 2023; Wrightson et al., 2020). Previous neuroimaging and behavioral studies suggest that locomotor control relies on distributed cortical, cerebellar, basal ganglia, and spinal mechanisms capable of maintaining rhythmic stepping with relatively limited executive oversight under predictable environmental conditions (Takakusaki, 2017). Rather than indicating an absence of cognitive-motor interaction, the present findings suggest instead that our conditions allowed for reserve capacity. Greater cognitive interference may become more evident under conditions involving environmental uncertainty, obstacle negotiation, balance perturbations, or irregular terrain rather than steady-state treadmill walking, especially for younger adults who were well within their physical walking capacity (Lee and Lee, 2023; Persad et al., 2008).

Although cognitive load exerted relatively modest effects on gait, age differences emerged consistently across several kinematic variables, some improving postural stability while others reduce it. Compared to younger adults, older adults demonstrated lower toe clearance, shorter relative stride lengths, greater stride frequencies, and increased time spent in double support throughout the experiment. These characteristics are consistent with conservative gait patterns generally reported in healthy aging studies that prioritize stability (Maki, 1997; Mills et al., 2008; Santhiranayagam et al., 2015; Winter et al., 1990; Menz et al., 2003). Increasing double support time increases the proportion of the gait cycle during which both feet remain in contact with the ground, effectively increasing mechanical stability. These general age-related differences reflect adaptive compensatory strategies that reduce fall risk. These adaptations are similar to those proposed in the compensation-related utilization of neural circuits hypothesis (CRUNCH; Reuter-Lorenz and Cappell, 2008), whereby older adults recruit additional neural resources at relatively low task demands to preserve behavioral performance. The present kinematic findings extend this concept to walking. Older adults appear to begin dual-task walking from a more conservative baseline strategy, potentially providing greater stability before additional cognitive demands arise. It is important to note that the conservative gait pattern refers to individuals who are able to decrease their walking speed when performing a cognitive task. In our study, participants were constrained to the same speed thus, older adults who adopted a shorter stride length had to also increase their stride frequency, which does not favor increased stability and is likely associated with lower walking economy. Dual-tasking increases energetic demand, particularly in older adults, consistent with evidence that cognitive-motor interference alters neuromuscular coordination and increases the metabolic cost of walking (Wu et al., 2026). The additional energetic demands imposed by increasing incline may have further challenged older adults’ ability to adapt their gait, suggesting that they may have had limited capacity to compensate for the combined cognitive and mechanical demands.

### Influences of cognitive performance, processing speed, and age on kinematics

Finally, executive function (or working memory) measures of RT and d′ from the *n*-back task, inform us about how cognitive abilities affected kinematic performance. Two broad patterns emerged: (1) age-related gait differences were present, but they were parameter-specific, concentrated in stride length, stride frequency, and toe clearance rather than reflecting a uniform age-related decline across all gait measures; and (2) RT and *d*′ are related to gait in qualitatively different ways—cognitive response speed was generally associated with gait parameters, while *d*′ was highly specific to incline, and in one case, task difficulty.

Age-related gait adaptation is parameter-specific, not global. Older adults showed a robust pattern of shorter relative stride length and higher stride frequency than younger adults, particularly at the highest incline for stride length. This compensation was not simply a byproduct of older adults’ slower preferred walking speeds: the age effect on stride frequency was larger after controlling for speed, indicating older adults adopt a higher-frequency gait despite their slower self-selected pace. Double support time and step width showed no meaningful age-related differences, and toe clearance showed only a modest overall age effect that did not vary by incline. This suggests that age-related adaptation under motor demand is concentrated on how far and how often the foot moves (stride length and stride frequency) rather than the how long the foot stays on the ground, how wide the steps are or vertical foot clearance (double support, step width, and toe clearance).

A central finding of this study is the dissociation between how RT and *d*′ are related to gait. RT showed a general, largely context-independent relationship with both relative stride length and stride frequency: slower responders showed longer relative strides and lower stride frequency, and this relationship largely held regardless of age group, incline, or *n*-back difficulty. This pattern is consistent with RT reflecting a relatively stable coupling between processing speed and gait conservatism—participants who respond more slowly under dual-task conditions may generally adopt a more deliberate, slower gait pattern, independent of the specific demands of the walking or cognitive task at hand.

*D*′, associated with executive function, had more specific effects. People who have better working memory performance (i.e., higher *d*′ scores) had shorter strides at the level incline but longer strides at the intermediate inclines. People with greater *d*′ also had lower step frequency at intermediate inclines and higher step frequency at the greatest cognitive load condition (2-back). *D*′ affected toe clearance: greater sensitivity led to greater toe clearance, but only at the level incline. Again, this is consistent with a greater chance of tripping as cognitive reserve is reduced. Double support was not affected by cognitive task performance, either *d*′ or RT. What is noteworthy is that these effects were related to executive function beyond any effect of age *per se*. Further, *d*′ reflects a context-dependent trade-off that varies according to the specific combination of physical and cognitive demand. It appears that *d*′ indexes a more effortful, resource-limited process than RT.

In sum, age explained average differences between groups, but executive function further explained how individuals responded to increasing dual-task demands. These findings are relevant to emerging work suggesting that executive function predicts gait impairment and fall risk independently of age (Montero-Odasso et al., 2002; 2012; Mirelman et al., 2012). Rather than viewing gait decline as an inevitable consequence of aging, the present findings support a model in which variability in executive function determines an individual’s capacity to successfully coordinate cognition and locomotion.

### Expanding on the posture-first hypothesis

Our results support a modified posture-first strategy with increased combined cognitive and motor demands. Behavioral performance clearly declined as cognitive load increased, whereas gait stability was largely maintained as kinematics adjusted to increased inclines. Older adults demonstrated reduced working memory sensitivity while maintaining conservative gait strategies characterized by greater double support and shorter strides. This pattern suggests that participants preferentially protected gait stability even when cognitive performance suffered. This interpretation aligns with the posture-first principle (Shumway-Cook and Woollacott, 2000) in that older adults prioritize stability when attentional resources become limited. However, the present findings refine this theory. Rather than observing breakdowns in gait, participants appeared to reallocate resources toward gait stability as demands increased, accepting declines in cognitive performance to preserve safe walking. Nonetheless, those with the greatest declines in cognitive performance could reallocate resources less efficiently, showing greater cognitive-motor interference. Of course, it is possible that relatively preserved average gait characteristics do not necessarily provide a complete picture of underlying motor or postural regulation such that average spatiotemporal measures may remain relatively stable while more subtle alterations in variability, dynamic stability, or movement regulation occur under increased task demands (Hausdorff, 2005, 2009; Lord et al., 2011).

### Future directions

This study suggests some potential future research directions. First, to investigate dual-task performance where participants were less able to switch between tasks, we required participants to walk on a treadmill to keep them walking even when the cognitive task became challenging. However, treadmill walking provides highly predictable walking conditions that reduce environmental uncertainty relative to overground walking. The constrained nature of treadmill walking, in addition to restricted motor difficulty in terms of speed and incline, may partially explain the relatively modest cognitive-load effects on gait, particularly for step width, which is mechanically constrained during treadmill walking. Further, requiring participants to maintain a constant speed as incline increased altered typical step frequency and relative stride length adjustments for increased incline that are typically accompanied by reductions in speed. Future studies should examine whether similar patterns emerge during obstacle negotiation, uneven terrain, or externally perturbed walking. Second, participants were healthy community-dwelling older adults who were capable of walking on a treadmill. Individuals with physical limitations, mild cognitive impairment, Parkinson’s disease, peripheral neuropathy, or a history of falls may exhibit substantially different patterns of cognitive-motor interaction. Third, combining the present paradigm with mobile EEG or functional near-infrared spectroscopy would allow direct examination of how cortical recruitment changes as cognitive and motor demands increase. Such approaches could determine whether individuals exhibiting larger cognitive costs require greater prefrontal recruitment to maintain gait stability, consistent with compensatory aging theories, such as CRUNCH and HAROLD (e.g., Cabeza, 2002; Park and Reuter-Lorenz, 2009). Additional measures could also include metabolic rate that could clarify energy expenditure during the task. Finally, including *d*′ and RT as independent predictors revealed relationships that would not be seen in traditional age-group analyses. Future work might treat executive function as a continuous individual-difference variable rather than relying solely on chronological age, as this approach may better capture the mechanisms underlying successful cognitive-motor integration.

## Conclusion

This study demonstrates that increasing executive and motor demands produce combined effects on working memory performance for older adults, while leaving gait surprisingly resilient in healthy adults. Rather than exhibiting widespread dual-task interference, older adults maintained gait stability by adopting conservative gait strategies characterized by shorter strides, greater percentage time in double support, and reduced adaptability to increasing motor demands. However, a key finding of this study was that individual differences in executive function and processing speed predicted gait adaptations in addition to observed changes with chronological age. These results suggest that successful cognitive-motor integration depends on the efficiency with which executive resources are allocated during locomotion, beyond any effects of aging. Consequently, measures of cognitive performance during dual-task walking may provide a sensitive behavioral marker of reduced locomotor reserve and may ultimately prove useful for identifying individuals at increased risk for future mobility decline and falls.

## Data Availability

The raw data supporting the conclusions of this article will be made available by the authors, without undue reservation.
