# Supplementary material for: Effects of age in a cognitive-motor dual task: parametric manipulations of cognitive load and motor difficulty
Source: Front Hum Neurosci. 2026 Sep 23;20:1927089. doi: 10.3389/fnhum.2026.1927089 (PMC13642604; doi:10.3389/fnhum.2026.1927089)
Supplement: Supplementary file 1 [file Table_1.docx]

Supplement A

Possible task orders all starting with performing the 0-back task at the level (0⁰) incline.

|  |  |  |  | **Session** |  |  |  |  |  |
| --- | --- | --- | --- | --- | --- | --- | --- | --- | --- |
|  | **1** | **2** | **3** | **4** | **5** | **6** | **7** | **8** | **9** |
| **Order** |  |  |  |  |  |  |  |  |  |
| 1 | A0/0B | A0/1B | A0/2B | A1/0B | A1/1B | A1/2B | A2/0B | A2/1B | A2/2B |
| 2 | A0/0B | A0/2B | A0/1B | A2/0B | A2/2B | A2/1B | A1/0B | A1/2B | A1/1B |
| 3 | A0/0B | A0/1B | A0/2B | A1/1B | A1/0B | A1/2B | A2/1B | A2/0B | A2/2B |
| 4 | A0/0B | A0/2B | A0/1B | A2/1B | A2/2B | A2/0B | A1/1B | A1/2B | A1/0B |
| 5 | A0/0B | A0/1B | A0/2B | A1/2B | A1/0B | A1/1B | A2/2B | A2/0B | A2/1B |
| 6 | A0/0B | A0/2B | A0/1B | A2/2B | A2/1B | A2/0B | A1/2B | A1/1B | A2/0B |

Incline Angles: A0 = 0-degree, A1 = 2.3-degree, A2 = 4.6-degree

Tasks: 0B = 0-Back, 1B = 1-Back, 2B = 2-Back
