# Supplementary material for: Effects of age in a cognitive-motor dual task: parametric manipulations of cognitive load and motor difficulty
Source: Front Hum Neurosci. 2026 Sep 23;20:1927089. doi: 10.3389/fnhum.2026.1927089 (PMC13642604; doi:10.3389/fnhum.2026.1927089)
Supplement: Supplementary file 2 [file Table_2.docx]

Supplement B

Principal effects (raw p < .05) and Benjamini-Hochberg (BH) false discovery rate correction

| **Outcome** | **Predictor** | **Term type** | **Raw p** | **BH-adjusted p (full family)** | **BH-adjusted p (within outcome)** |
| --- | --- | --- | --- | --- | --- |
| Stride length | Motor Difficulty | Main effect | <.001 | <.001 | <.001 |
| Stride length | Speed | Main effect | <.001 | <.001 | <.001 |
| Stride length | Group × Incline | Interaction | .0023 | .0099 | .0090 |
| Stride length | RT | Main effect | .0107 | .0261 | .0321 |
| Stride length | RT × Motor Difficulty × Group | 3-way interaction (exploratory) | .0344 | .0705 | .0825 |
| Stride frequency | Group | Main effect | <.001 | .0029 | .0052 |
| Stride frequency | Group × Motor Difficulty | Interaction | .0040 | .0135 | .0148 |
| Stride frequency | RT × Motor Difficulty × Group | 3-way interaction (confirmed) | .0049 | .0145 | .0148 |
| Double support | Group | Main effect | .0024 | .0099 | .0056 |
| Double support | Motor Difficulty | Main effect | <.001 | <.001 | <.001 |
| Double support | Cognitive Load | Main effect | .0087 | .0237 | .0152 |
| Double support | Speed | Main effect | <.001 | <.001 | <.001 |
| Step width | Motor Difficulty | Main effect | .0034 | .0127 | .0142 |
| Step width | d′ | Main effect | .0234 | .0532 | .0467 |
| Step width | d′ × Motor Difficulty | Interaction | .0047 | .0145 | .0142 |
| Toe clearance | Group | Main effect | <.001 | <.001 | <.001 |
| Toe clearance | Motor Difficulty | Main effect | <.001 | <.001 | <.001 |
| Toe clearance | d′ | Main effect | .0108 | .0261 | .0190 |
| Toe clearance | d′ × Motor Difficulty | Interaction | .0002 | .0013 | .0005 |

*Note. Benjamini-Hochberg correction was applied both across the full set of principal effects reported across all five gait outcomes ("full family") and separately within each outcome ("within outcome"). Rows shaded in red indicate effects that did not survive BH correction under at least one approach.*
